# *RNA-Seq* reveals divergent gene expression between larvae with contrasting trophic modes in the poecilogonous polychaete *Boccardia wellingtonensis*

**DOI:** 10.1038/s41598-021-94646-y

**Published:** 2021-07-22

**Authors:** Álvaro Figueroa, Antonio Brante, Leyla Cárdenas

**Affiliations:** 1 Instituto de Ciencias Ambientales y Evolutivas, Universidad Austral de Chile, Valdivia, Chile; 2 Facultad de Ciencias, Centro de Investigación en Biodiversidad y Ambientes Sustentables, Universidad Católica de la Ssma, Concepción, Concepción, Chile; 3 Departamento Ecología, Facultad de Ciencias, Universidad Católica de la Santísima Concepción, Concepción, Chile; 4 Centro Fondap-IDEAL, Valdivia, Chile

**Keywords:** Evolutionary developmental biology, Gene expression

## Abstract

The polychaete *Boccardia wellingtonensis* is a poecilogonous species that produces different larval types. Females may lay Type I capsules, in which only planktotrophic larvae are present, or Type III capsules that contain planktotrophic and adelphophagic larvae as well as nurse eggs. While planktotrophic larvae do not feed during encapsulation, adelphophagic larvae develop by feeding on nurse eggs and on other larvae inside the capsules and hatch at the juvenile stage. Previous works have not found differences in the morphology between the two larval types; thus, the factors explaining contrasting feeding abilities in larvae of this species are still unknown. In this paper, we use a transcriptomic approach to study the cellular and genetic mechanisms underlying the different larval trophic modes of *B. wellingtonensis.* By using approximately 624 million high-quality reads, we assemble the de novo transcriptome with 133,314 contigs, coding 32,390 putative proteins. We identify 5221 genes that are up-regulated in larval stages compared to their expression in adult individuals. The genetic expression profile differed between larval trophic modes, with genes involved in lipid metabolism and chaetogenesis over expressed in planktotrophic larvae. In contrast, up-regulated genes in adelphophagic larvae were associated with DNA replication and mRNA synthesis.

## Introduction

Marine invertebrates exhibit contrasting developmental modes that may affect the speciation, extinction, and connectivity of species^1^. In species that encapsulate their offspring, the indirect developmental mode is characterized by embryos that develop partially inside capsules and hatch as planktotrophic larvae. This type of larvae shows a set of morphological traits that allows them to swim and feed on plankton. At the other end of the spectrum, species with direct development complete the whole larval development inside the capsule and hatch at the juvenile stage^1–3^. Within the direct development mode, larvae may show different intracapsular feeding strategies: lecitotrophy, in which larvae use an internal energetic source provided in the egg by way of yolk (lecitotrophic larvae); ovophagy, in which larvae feed on nurse eggs provided by the mother (ovophagic larvae); and adelphophagy, in which larvae feed on other embryos (adelphophagic larvae)^2^. From an ecological perspective, lecithotrophy and adelphophagy appear to be advantageous strategies due to the larger resulting hatching sizes, higher survival rates, and better competitive abilities of the hatchers^3–5^. On the other hand, planktotrophy allows a potentially greater larval dispersion range and the colonization of new habitats, thus reducing resource competition^6^.

The different nutritional strategies of the larval trophic modes have been associated with morphological evolutionary transitions, and in most marine invertebrate species they are considered taxonomic traits^7,8^. However, in some taxonomic groups with larval encapsulation, such as polychaetes and gastropods, the production of different larval types in the same species has been documented^9^. This reproductive strategy, called poecilogony, is a rare but interesting characteristic that depending on the species, different larval trophic modes can be observed within or between capsules, clutches, or populations; additionally, females may produce different larval types along the time. For example, in the poecilogonous polychaetes *Boccardia wellingtonensis* and *Boccardia proboscidea,* females lay both planktotrophic and adelphophagic larvae in the same clutch or capsule. These larvae are morphologically similar but differ in their feeding capacities; while both larval types can swim and feed on plankton if they are released into the water, only adelphophagic larvae may feed on nurse eggs and other larvae inside the capsules^10,11^. This suggests the existence of underlying mechanisms, other than morphological traits, that would explain the feeding behavior and capacity of marine invertebrate larvae. In this way, marine species that exhibit poecilogony provide an interesting biological model to study the evolutionary transitions between the different larval trophic strategies as well as the developmental modes in marine invertebrates because they enable intraspecific comparisons without underlying phylogenetic effects^12^.

The poecilogonous polychaete *Boccardia wellingtonensis* inhabits the intertidal zones of Chile, South Africa, and New Zealand. Females of this species reproduce throughout the year laying capsules that resemble the shape of a pearl necklace, which intensifies sibling competition for food at the larval stage. Oyarzun and Brante^10^ describe two reproductive strategies in this species: Type I females produce only planktotrophic larvae while Type III females produce capsules containing both planktotrophic and adelphophagic larvae, which are indistinguishable from one another in the early stages of development, as well as nurse eggs. While planktotrophic larvae stop growth at mid developmental stages and do not appear to feed inside their capsules, adelphophagic larvae feed on nurse eggs and other embryos until the offspring hatch at an advanced larval stage or as juveniles^10^ (Supplementary File S5). Previous works have found differences in the enzymatic activity of some digestive enzymes, such as hydrolases, hydrolases acids, and glycosidase, in the larvae of *B. wellingtonensis* that could be associated with the different larval trophic modes observed in this species^13^. Moreover, differences in growth rates of isolated embryos in vitro^10^ and the differing expression of microRNAs between both types of larvae^14^ suggest that the genetic mechanisms operating during early development could be driven trophic mode evolution in poecilogonous species.

Differences in gene expression profiles have been associated with contrasting early developmental patterns in marine invertebrates. For example, in echinoderms, transcriptomic analyses have expanded the knowledge of the cellular mechanisms responsible for developmental processes, for which a Gene Regulatory Network (GRN) has been described in detail^15,16^. The determination of GRNs has revealed differences in the expression of genes with known roles in skeletal, endomesoderm, and ectoderm control in different species with planktotrophic and lecithotrophic development^17^. Likewise, significant differences have been identified in gene expression in the early developmental stages between planktotrophic *Clypeaster subdepressus* larvae and facultative planktotrophic *C. rosaceus* larvae^18^. In the case of the poecilogonous polychaete *Pygospio elegans*, the gene-expression profiles of the two types of females, planktotrophic and benthic, have a reproductive bias^19^. Between the two types, more than 8000 differentially expressed unigenes have been identified. While benthic females were enriched in the transcription of genes associated with extracellular protein production including collagens, vitrin, and fibropellin-1, planktotrophic females showed enrichment in genes associated with metabolism and intracellular proteins^19^. From an evolutionary perspective, the plasticity in the aforementioned gene expression suggests its potential relationship with the evolutionary transition of different larval trophic modes. In this framework, exploration of these potential underlying mechanisms in *B. wellingtonensis* would improve the existing understanding of evolution and the diversity of developmental modes in marine invertebrates.

Here, we study the molecular mechanisms that could determine the different larval trophic modes in *Boccardia wellingtonensis* through the first transcriptome for this species developed using the RNA-seq approach*.* Transcriptomes were obtained from: (1) males, (2) females showing Type I and Type III reproductive strategies, (3) Type I planktotrophic larvae, (4) Type III planktotrophic larvae, (5) early adelphophagic larvae, and (6) late adelphophagic larvae. Knowing the transcriptome allows us to correctly interpret the functional elements of the genome and uncover molecular factors at the cellular, tissue, and individual levels^20^. From that point of view, in this work we examine whether transcriptional changes could explain the diversity of larval trophic modes in *B. wellingtonensis.*

## Results

### De novo transcriptome assembly and quality assessment

We sequenced the RNA libraries of seven sample groups of *B. wellingtonensis* individuals including three groups of adults: Type I females (FTI), Type III females (FTIII), and males (M); and four groups of larvae: Type I planktotrophic larvae (PLTI), Type III planktotrophic larvae (PLTIII), early adelphophagic larvae (EAL), and late adelphophagic larvae (AL). Each group had three biological replicates. The total of 21 libraries produced a total of 1,157,058,616 PE reads with an average of 55.1 ± 6.2 (mean ± SD) million reads per sample. The raw reads were subjected to a quality trim (Phred = 0.001), and adapters were removed, resulting in a total of 624,918,240 PE post-trim reads with an average of 85.04 bp (Supplementary File S1). The trimmed reads were subjected to a de novo assembly using CLC Genomics, which produced 172,381 transcripts. To reduce the number of potentially spurious transcripts, we filtered based on the abundance and quality of the assembly. To do so, we used the Salmon selective alignment method (–validateMappings) to detect the levels of expression and redundancies. In the end, 133,314 highly expressed transcripts were kept with an N50 = 1532 bp, a % CG = 46.82 and size = 79 Mb. The longest transcript contains a sequence of 28,098 bp, and 18,805 transcripts measure more than 1 Kb. The length and distribution of all of the assembled transcripts are shown in Supplementary File S2. Meanwhile, a BUSCO (Universal Single‐Copy Orthologs) analysis revealed a high rate of 862 (88.14%) orthologous genes from the Metazoa_odb9 database with few fragmented or missing BUSCO (Complete gene representation: 76.69% [Single copy: 73.21%, Duplicated: 3.48%], Fragmented: 11.45%, Missing: 11.86%), indicating a high-quality transcriptome. A summary of the assembly of the transcriptome and a quality assessment are shown in Table 1.

**Table 1:** Summary of the transcriptome assembly and quality assessment of *B. wellingtonensis.*

Number of raw PE reads	1,157,058,616
High quality PE reads	624,918,240
Transcripts	172,381
Transcripts after filter	133,314
Assembly size (MBP)	79
GC content	46.82
N50 (BP)	1532
N75 (BP)	909
L50 (BP)	10,022
L75 (BP)	20,893
Largest contig	28,098
Transcripts > 1 KB	18,805
BUSCO completeness (%)	88.14

### Functional annotation and gene ontology mapping

**Figure 1:**
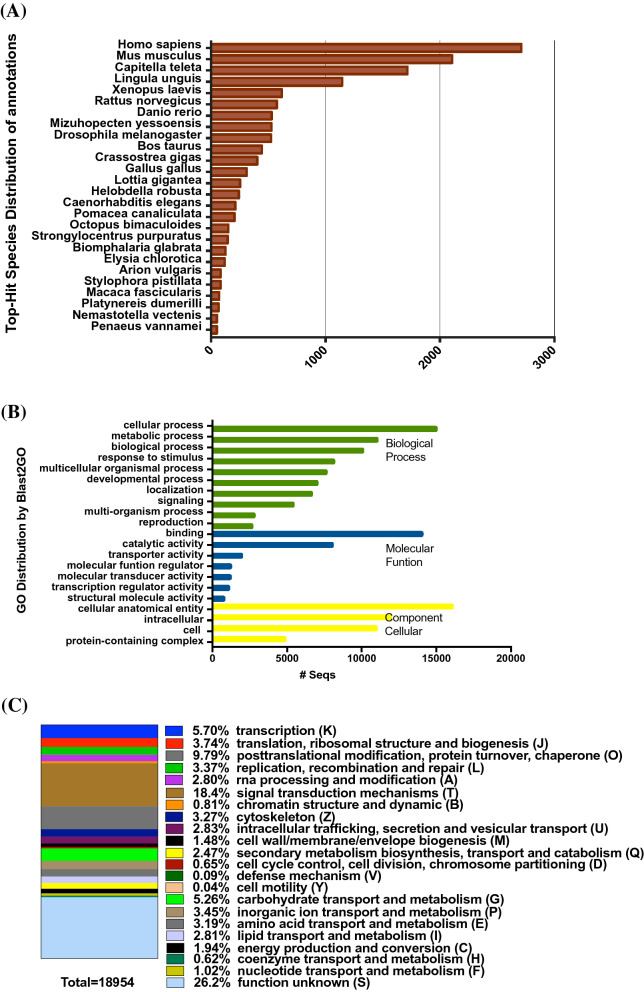
Characteristics of genes. (**A**) Top ten species distribution. (**B**) Functional annotation based on Gene Ontology (GO) categorization. (**C**) COG categories distribution of EggNOG classifications.

The assembly of the filtered transcripts of *B. wellingtonensis* were subject to the identification of coding sequences (CDS) using Transdecore. A total of 32,390 transcripts contained an open reading frame (ORF) of at least 100 aa in their sequences. The databases Uniref90 and UniProtKB were used in Blastx to search for homologs of the transcripts coded as proteins. In Uniref90 (blastx e-value cutoff 1.0E-10), the search resulted in 9,792 annotated transcripts, but they included a high number (2,401) of uncharacterized Lophotrochozoa proteins. For that reason, we ran the CDS against the entire UniprotKB database to obtain functional annotations. We found that 16,158 transcripts were annotated with the UniprotKB database (blastx e-value cutoff 1.0E-06). We merged both annotation tables using Blast2GO, which resulted in 19,277 annotated transcripts. After discarding the terms ‘strain’, ‘viridae’, ‘hypothetical’, ‘uncharacterized’, and ‘unknown’, the resulting database of annotated genes contained 16,765 annotated transcripts. The distribution of species with the most hits (Fig. 1A) was dominated primarily by *Homo sapiens* with 2713 hits, followed by *Mus musculus* with 2107 hits, and *Capitella teleta* with 1715 hits. The marine species with the most hits were invertebrates such as *Lingula unigis*, *Mizuhopecten yessoensis*, *Cassotrea gigas,* and *Lottia gigantea.*

The GO (gene ontology) classifications were obtained from the results of the annotations in Uniref90, UniProt, and InterProScan using Blast2GO. A total of 247,996 Level 2 GO assignments were generated from the annotations, and they were sorted into three GO domains, which include biological processes, molecular functions, and cellular components (Fig. 1B). In the biological process category, the most frequent assignments were cellular process (14,965), metabolic process (11,006), and biological regulation (10,137). Other significant assignments were response to stimulus, multicellular organismal process, and developmental process (Fig. 1B). The domains of the molecular function were strongly dominated primarily by binding (14,010) and catalytic activity (8003) (Fig. 1B). In the domains of cellular components, the annotated transcripts were assigned mainly to cellular anatomical entity (16,041), followed by intracellular (11,889), and cell (10,494) (Fig. 1B). The annotation made in EggNOG resulted in 13,068 assignments (Fig. 1C). The assignments were sorted into 23 categories, of which Unknown Function (S) (4971) was the most prominent, followed by signal transduction mechanism (T) (3497), posttranslational modification, protein turnover, chaperones (O) (1855), transcription (K) (1081), carbohydrate transport and metabolism (G) (997), translation, ribosomal structure and biogenesis (I) (708), inorganic ion transport and metabolism (P) (654), among others.

### Differential expression analysis

**Figure 2:**
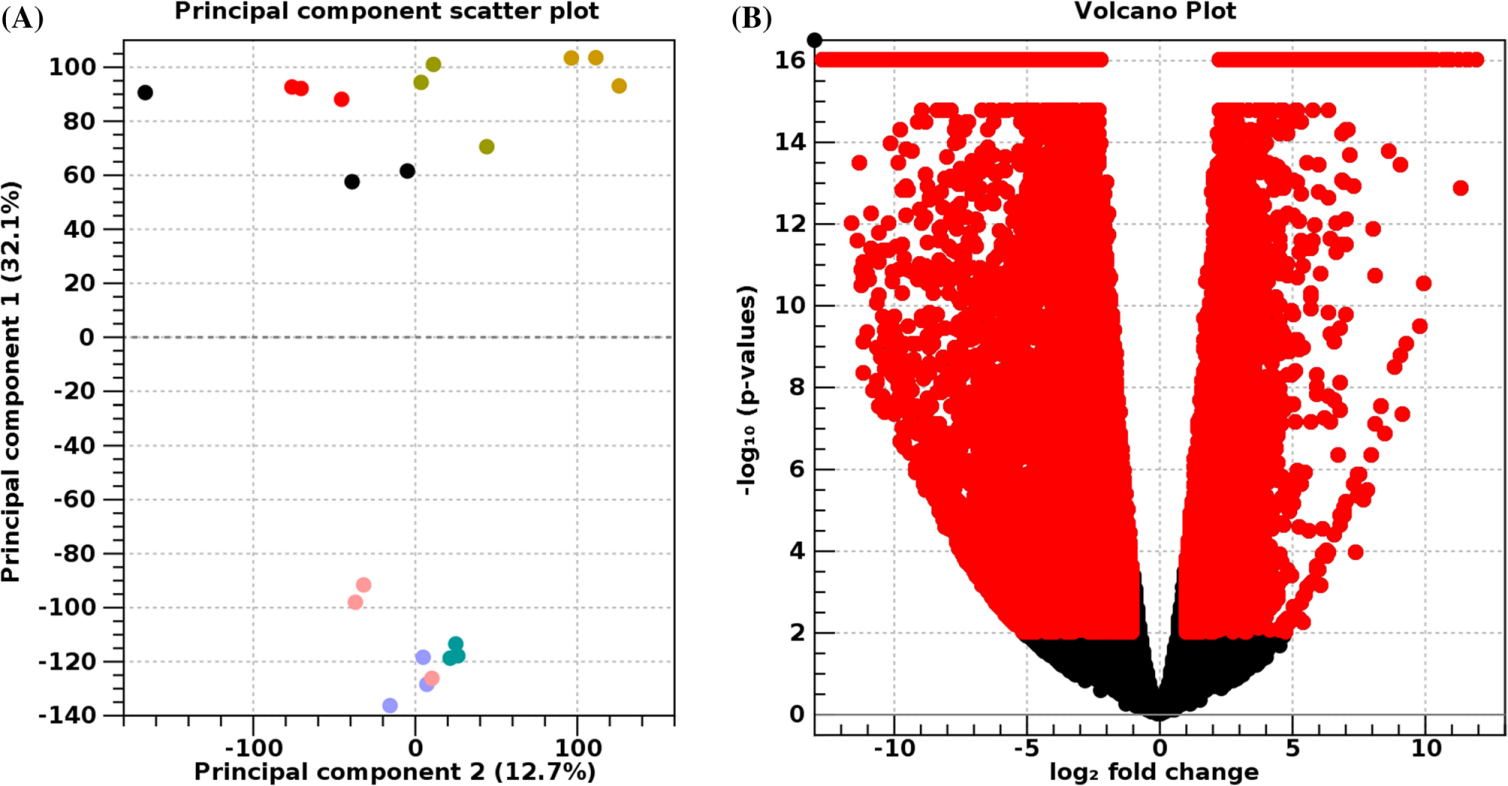
Adult individuals and larval state of *B. wellingtonensis* show a unique transcriptional signature. (**A**) Principal component analysis of the 21 RNAseq sample shows clustering into adult and larval groups. Upper circles show larval samples: EAL (red), AL (black), PLTIII (green) and PLTI (yellow). Lower circles show adult samples: FTIII (pink), FTI (lilac), and M (calypso). (**B**) Volcano plot comparison of differential gene expression between larvae and adults. Red dots highlight differentially expressed genes with threshold FDR < 0.01 and Log2 Fold Change [≥ 1]. The black zone indicates the number of transcripts that do not show significant differential expression.

To compare the expression patterns among adults, larval trophic modes and developmental stages in *B. wellingtonensis*, a principal component analysis (PCA) was performed on the normalized transcripts (Fig. 2A). The PCA showed that the adult and larval samples grouped separately within principal component 1 (PC1) with a 32.1% of the variance explained by this component (Fig. 2A). It should be noted that one of the AL replicates (AL1) showed a lower number of reads in comparison to the other samples resulting in an outlier sample, which could affect the statistical accuracy of the whole dataset (Supplementary File S1). Consequently, for further analyses, the AL samples were not included.

To identify up-regulated genes, we first compared the whole dataset; i.e., adults (FTI, FTIII, and M) versus larval stages (EAL, PLTI, and PLTIII). We identified 13,421 transcripts that exhibit differential expression (FDR < 0.01; Log2 Fold Change [> 1]) of which 5,221 are up-regulated in larvae while 8,200 are up-regulated in adults (Fig. 2B). The top ten differentially expressed genes (DEGs) in these comparisons are described in Table 2. Next, a comparison between adult females (FTI vs. FTIII) identified 374 DEGs (FDR < 0.01; Log2 Fold Change [> 1]; 128 up-regulated in FTI and 246 up-regulated in FTIII) (Supplementary File S4). Then, pairwise comparisons among larval trophic modes (PLTI vs. EAL and PLTIII vs. EAL) identified 8,670 DEGs between PLTI and EAL (FDR < 0.01; Log2 Fold Change [> 1]; 5,065 up-regulated in PLTI and 4,862 up-regulated in EAL) (Supplementary File S3A). The comparison between PLTIII and EAL identified 966 DEGs (FDR < 0.01; Log2 Fold Change [> 1]; 455 up-regulated in PLTIII and 511 up-regulated in EAL) (Supplementary File S3B). The Venn diagram identified 378 DEGs that overlapped in both planktotrophic groups (PLTI and PLTIII) (Supplementary File S3C). The top ten DEGs in these comparisons are described in Table 2. The complete list of DEGs with their functional annotations in the different expression analyses are listed in Supplementary File S4.

**Table 2:** The top ten up- and down-regulated genes of differential expression analysis.

Transcript	Annotation	Log2FC	FDR*	Transcript	Annotation	Log2FC	FDR
**Larvae versus adults (control)**							
contig_121728	ShkT	11.69	0.00	contig_38067	CILP1	− 12.69	0.00
contig_122231	APOD	11.62	0.00	contig_995	HMCN1	− 12.56	0.00
contig_25299	DRK	9.95	0.00	contig_938	VIT6	− 12.04	0.00
contig_37708	GHF71	9.87	0.00	contig_775	THYG	− 11.61	0.00
contig_944	PPIA	9.56	0.00	contig_3	Amine oxidase	− 11.52	0.00
contig_87606	CBPA1	9.21	0.00	contig_5742	VKT1	− 11.51	0.00
contig_8969	GDIR1	9.14	0.00	contig_1453	AGRB1	− 11.38	0.00
contig_88814	NOTC1	9.02	0.00	contig_276	F5/8TCP	− 11.31	0.00
contig_121973	JAG1A	8.91	0.00	contig_1381	PIF	− 11.23	0.00
contig_122091	NOTC3	8.17	0.00	contig_1999	BMPH	− 11.18	0.00
**PLTI versus EAL (control)**							
contig_134018	CYP 450 3A24	6.48	0.00	contig_27408	NR1D1	− 6.85	0.00
contig_82481	COEA1	6.03	0.00	contig_112201	METTL27	− 6.81	0.00
contig_111772	FAT2	5.78	0.00	contig_114466	ALPL	− 6.35	0.00
contig_49126	DLL1	5.38	0.00	contig_22998	ADAT1	− 4.89	0.00
contig_124622	NNMT	5.20	0.00	contig_36302	Her9	− 4.84	0.00
contig_122297	TRPM8	5.06	0.00	contig_82796	THB	− 4.67	0.00
contig_82597	ZAN	5.04	0.00	contig_57569	HES1	− 4.37	0.00
contig_60913	LOTGIDRAFT_174331	4.72	0.00	contig_18774	ARRH	− 4.16	0.00
contig_74887	TMEM8A	4.16	0.00	contig_8396	PRI1	− 4.14	0.00
contig_85773	DYH1	4.11	0.00	contig_94926	SLC39A4	− 4.07	0.00
**PLTIII versus EAL (control)**							
contig_7950	Sulfotransfer	2.94	3E−11	contig_75954	PAP17	− 3.46	0.00
contig_42211	PLB1	2.30	6E−10	contig_25226	APEX1	− 1.82	3E−08
contig_76672	Lipasa_GDSL	2.35	5E−10	contig_36302	Her9	− 2.52	2E−07
contig_397	Desaturase	2.04	9E−09	contig_8369	PRI1	− 1.62	3E−06
contig_73820	COMP	1.75	6E−08	contig_35950	DPOLB	− 1.59	5E−06
contig_122231	APOD	2.19	3E−08	contig_43699	LOC106173161	− 1.63	3E−06
contig_13435	CGI_10019207	2.00	5E−07	contig_57569	HES1	− 2.44	3E−06
contig_99037	CES2	2.29	5E−07	contig_53940	ASNS	− 1.62	6E−05
contig_46413	CCF1	1.77	5E−07	contig_51986	I206_04028	− 1.81	3E−05
contig_29812	PKD1	2.34	6E−06	contig_84733	CEL2A	− 2.32	1E−05
**FTI versus FTIII (control)**							
contig_90	C1QT6	3.84	6E−09	contig_112532	Deoxyrribonucleasa	− 9.46	0.00
contig_8142	DHX33	4.86	9E−08	contig_9082	Tetraspanin	− 9.36	0.00
contig_4708	RRBP1	5.34	2E−07	contig_7012	SLC3	− 9.34	0.00
contig_10208	VP13D	3.65	6E−07	contig_12667	GGT1	− 6.88	0.00
contig_34256	IFI44L	4.22	8E−07	contig_177	PZP	− 5.94	0.00
contig_19648	ADRO	4.82	1E−06	contig_27382	RR_TM4-6	− 8.95	7E−13
contig_34428	GRID1	8.18	2E−06	contig_164419	ITB7	− 8.98	1E−12
contig_14667	hexosyltransferase	3.16	3E−06	contig_128776	LORF2	− 5.11	1E−08
contig_9006	AT133	2.67	8E−06	contig_39099	GGT	− 5.17	4E−08
contig_4285	KLH38	4.56	2E−05	Contig_2882	SPARC	− 6.79	8E−08

*FDR = 0.00 indicates values of 1E−16 or lower.

### GO enrichment analysis

**Figure 3:**
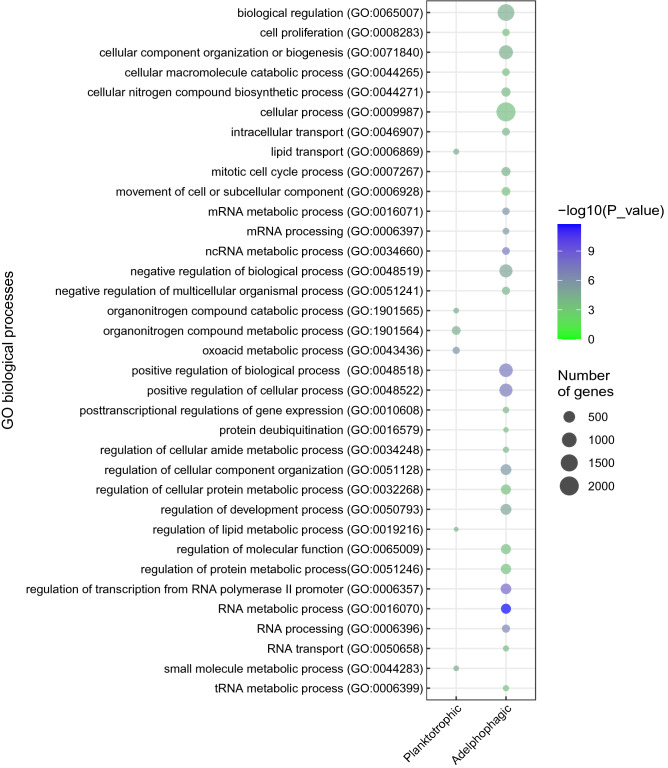
Plot of the enriched biological processes (FDR < 0.05) identified in adults, larval stages, planktotrophic (PLTI and PLTIII) and adelphophagic (EAL) larvae. The GO terms were collapsed in REVIGO. The size of the dot indicates the number of genes grouped in a GO term, and the color represents the significant enrichment -Log10(FDR). The vertical line represents the number of DEGs.

A total of six GO terms were enriched in planktotrophic larvae (Type I and III) in biological processes (BP) (FDR < 0.05 and collapsing by REVIGO) (Fig. 3). These terms include the oxoacid metabolic process (GO:0043436) along with enriched processes related to lipid transport (GO:0006869) and the regulation of the lipid metabolic process (GO:0019216). The organonitrogen compound catabolic process (GO:1901565) and the organonitrogen compound metabolic process (GO:1901564) are also included in this group (Fig. 3). Eleven GO terms were found to be related to molecular functions (MF), and four were related to cellular components (CC). Details of the GO terms enriched in BP, MF, and CC are listed in Supplementary File S4. In adelphophagic larvae, 29 GO terms were related to biological processes (BP) (FDR < 0.05 and collapsing by REVIGO) (Fig. 3). Other noteworthy RNA terms include RNA transport (GO:0050658), regulation of transcription from RNA polymerase II promoter (GO:0043436), mRNA processing (GO:0006397), tRNA metabolic process (GO:0006399), ncRNA metabolic process (GO:0034660), and post-transcriptional regulation of gene expression (GO:0010608). Other relevant GO terms were regulation of developmental process (GO:0050793), cell proliferation (GO:0008283), and mitotic cell cycle process (GO:1903047) (Fig. 3). Twenty-one GO terms were found to be related to MF, and eleven were related to CC. Details of the GO terms enriched in BP, MF, and CC are listed in Supplementary File S4.

## Discussion

Here we assembled the first de novo transcriptome of the species *B. wellingtonensis*, a poecilogonous species, to characterize its genes and their expression profiles focused in the DEGs related to the different larval trophic modes. This study provides a new source of genetic information for research in polychaetes and reveals differences in the gene expression profiles among the different larval trophic modes. The results suggest that the regulation of gene expression could be a key mechanism underlying larval trophic strategies in the poecilogonous species. Early evidence suggests that the transcriptome profile could be associated with the developmental mode in poecilogonous species^19,21^. In our work, we use the recent development of sequencing technology to perform RNAseq experiments to test for the profile of differentially expressed genes of different individuals in a whole genome approach, which enables us to search for the genes particularly responsible for the evolution of their developmental modes.

### Comparison of DEGs in larvae compared to adults

Comparisons between the adult individuals and the larval stages (Fig. 2) identified DEGs in adults mainly related to physiological processes like reproduction, environmental responses, and the maintenance of the cellular matrix (Table 2). For example, we found an elevated expression of the vitellogenin 6 (VIT6), a the egg yolk precursor protein and for the transport of lipids; in females, it is a specific indicator of oocyte development^22^. Other up-regulated genes are CLIP1, HMCN1, VKT1, and ADGR1, which produce proteins for the extracellular matrix and are relevant to the integrity of the structural tissues^23^. In *Caenorhabditis elegans*, hemicentin (HMCN1) is an extracellular protein that forms regulatory networks in muscular and gonad tissues and facilitates the mechanosensory neurons’ anchorage to the epidermis^23^. In the polychaete *Schmidtea mediterránea,* hemicentin contributes to the correct proliferation of stem cells^24^. Another interesting finding is the elevated expression of thyroglobulin (THYG), a glycoprotein that assists in the synthesis of thyroid hormones; in invertebrates, it can act as a sensor of iodinated tyrosine, an indicator of food availability^25^.

In the larval state, we observed the overexpression of JAG1A, a protein related to the Notch pathway (Table 2), and components of the Notch pathway such as NOTC1 and NOTC3 (Table 2). Recently, it was reported that the Notch pathway in the annelid *Platynereis dumerilii* are expressed in the structures that give rise to the nascent chaetae and that deterioration causes malformations and abnormalities in chaetae-producing cells^26^. Therefore, the components of the Notch pathway in larvae of *B. wellingtonensis* could be related to chaetogenesis and/or to morphological development. Moreover, previous studies have found that the Notch pathway is involved in the regulation of settlement and metamorphosis of polychaetes^27^. In other annelids, such as *Capitella teleta* and *Hydroides elegans*, the segmentation process is controlled by Notch, the Wnt signaling pathway, and various Hes genes including Hes1^28^. In *B. wellingtonensis*, Hes1 and Wnt1 are up-regulated in the larval state (Supplementary File S4). These genes likely regulate the segmented growth in *B. wellingtonensis*^28^. However, the cumulative evidence does not point to a common mechanism that regulates the segmentation in all annelids^26^. Therefore, it seems necessary to better understand the molecular control of larval development during their intracapsular development and to analyze the temporality and the special expression of genes such as Hes1 and Wnt1 and the components of the Notch signaling pathway.

### Comparison of DEGs between adults

Low numbers of DEGs were detected in the comparison between females (FTI and FTIII). Knowledge of these genes’ functions is still poor, and it is therefore not easy to develop a clear picture of the relationships between the female developmental mode and egg production. In FTIII, high levels of expression in SLC3, GGT1, and ITB7 genes were detected. In the polychaete *Osedax japonicus*, a bone-eating worm, the transporter SLC plays a crucial role in nutrient absorption^29^. In the invertebrate chordate *Ciona intestinalis*, the Gamma-glutamyltransferase 1 (GGT1) is part of the core pathway of genes contributing to glutathione biosynthesis, an important metabolic mechanism in the specie’s environmental stress response^30^. In the sea cucumber *Apostichopus japonicus*, the integrin beta 7 (ITB7) is part of the family of cell adhesion molecules that plays an important role in a wide range of physiological processes including the immune response^31^.

### Comparison of DEGs between larvae

The comparative analyses between planktotrophic (PLTI, PLTIII) and adelphophagic (EAL) larvae suggest that the enrichment of DEGs observed in planktotrophic larvae is related to the regulation of the lipid metabolic process (GO:0019216) and lipid transport (GO:0006869) (Fig. 3). Lipids are the main reserve of metabolic energy in animals, and they play a central role in the larval development of marine invertebrates^32^. Of the total upregulated genes found in PLTI, four genes contain desaturase domains in PLTI: Protocadherin FAT2, Protocadherin FAT4, FAT atypical cadherin 4, and Fat acyl-CoA hydrolase medium chain (Fragment) (Table 2, Supplementary File S4). Desaturase enzymes are directly involved in the biosynthesis of polyunsaturated fatty acids (PUFA) in invertebrate marine animals^33^. The desaturases are widely distributed across animal phyla, and in fact, multiple invertebrates can actively produce PUFA de novo^34^. For example, the annelid *Riftia pachyptila* has been shown to have a desaturase that enables it to biosynthesize α-Linolenic acid^35^. In the specific case of, the FAT2 desaturase catalyzes the first step for the biosynthesis of PUFA^36^. Our data suggests that in planktotrophic larvae the desaturase enzymes can selectively control the PUFA metabolism and thus regulate lipid composition. Fourteen other strongly over-regulated genes are lipases and lipoxygenase domain-containing proteins. Interestingly, some genes that PLTI and PLTIII have in common, such as PLB1, Lipasa-GDSL, LIPP, and LOXH1, possess lipase domains that may, in combination with various transport lipoproteins, control the composition of structural lipids such as phospholipids and cholesterol. Based on our results, we hypothesize that in *B. wellingtonensis*, planktotrophic larvae should maximize the use of lipids as nutrition sources provided by the mother within the egg. It has been observed that in echinoderms with planktotrophic development, the utilization of lipids as a source of energy is dynamic and highly regulated in order to sustain the development of the egg, the growth, and the survival of the larvae to ensure successful settlement and subsequent metamorphosis^37^. Specifically, larvae use lipovitellin, a component found in the internal reserves of embryos and larvae that contains high levels of phospholipids in polychaetes^38^.

We observed more DEGs between PLTI and EAL than we did between PLTIII and EAL (Supplementary File S3A,B). In contrast to the congeneric species *B. proboscidea,* the Type III reproductive strategy in *B. wellingtonensis* is characterized by a wider range of larval sizes (PLTIII), which is probably influenced by intracapsular food availability^10^. This causes a high variance in the larval size, making it likely to find larvae in different developmental stages in the same capsule. We also hypothesized that maternal imprinting could be another important factor. To arrive at a final conclusion, more experiments should be done to determine which external factors influence the transcript expression during the species’ development. It is important to note that over 80% of the up-regulated DEGs in PLTIII are shared with PLTI (Supplementary File S3C)*.* Further investigation of this overlapped expression pattern of 378 genes up-regulated in planktotrophic larval in other Polydora-complex species could shed light on the molecular and genetic mechanisms explaining the diversity of larval traits and the evolution of developmental modes in marine invertebrates^39^*.*

In PLTI larvae, five up-regulated transcripts can be classified into the Cytochrome P450 family, with CYP 450 3A24 being one of the top DEGs (Table 2). The CYP 450 family plays a pivotal role in the physiology of marine invertebrates, particularly in annelids, by catalyzing the biosynthesis of signal molecules including steroids^40^. Further, in the embryos of mussels, these enzymes are important for the metabolism of xenobiotics such as carcinogens, pesticides, and drugs^41^. In particular, the expression of CYP 450 3A24 is associated with the palmitoleic acid-induced stress response in the *Argopecten irradians*^42^. Moreover, other multifunctional enzymes that detoxify the xenobiotics, such as the glutathione S-transferases (GST), are also expressed in PLTI’s DEGs, (Supplementary File S4). In this context, the up regulation of CYP450 and GST genes could be fundamental in PLTI’s response to environmental stress.

Another remarkable find is the presence of TRPM8 and several acid-sensing ion channels (ASICs) in PLTI (Table 2 and Supplementary File S4). TRPM8 is an important sensor in cold environments that requires the presence of phospholipids in the membrane^43^. In larvae of marine invertebrates, temperature regulates metabolism, growth rate, and the duration of the larval development^44^. ASICs belong to the family of degenerin/epithelial sodium channels, which are presumably involved in mechanosensation^45^. This mechanosensory response is key during feeding, tactic responses or predator–prey interactions in marine animals^46^. In general, our results suggest that genes with the highest expressions in planktotrophic larvae are related firstly to energy production from endogenous sources, primarily phospholipids, and secondly to the formation of structures and the sensory system that enable the capture of exogenous food to complete the plankton’s development.

Our analyses of EAL compared to planktotrophic larvae (PLTI, PLTIII) found significant increases in the number of GO terms related to: regulation of the developmental process (GO:0050793), cell proliferation (GO:0008283), cellular component organization or biogenesis (GO:0,071,840), cellular process (GO:0009987), and the mitotic cell cycle process (GO:0007267). Furthermore, RNA polymerase II promoter (GO:0006357), mRNA processing (GO:0006397), and mRNA metabolic process (GO:0016071) were also detected (Fig. 3). These results align with an adelphophagic development that, in comparison with planktotrophic development, is characterized by a rapid increase in size until hatching at the juvenile stage. Oyarzun and Brante^10^ explained that shortly after the first segment appears in adelphophagic larvae of *B. wellingtonensis*, larvae begin to feed on nurse eggs reaching an average length of 13.3 setigers after 15 days at 20 °C^10^. Studies in the nemertine *Lineus ruber* provided evidence that cells in a proliferative stage are widespread throughout fast adelphophagic development, from gastrulation until metamorphosis^47^. Cell proliferation is highly regulated by transcription factors that contain bHLH (basic helix-loop-helix) domains^48^. We observed a high expression of 37 transcription factors in the EAL of *B. wellingtonensis*; of them, five DEGs contained the bHLH domain (Supplementary File S4).

Meanwhile, another important group of 39 DEGs were related to DNA replication (Supplementary File S4). Nevertheless, despite the fast growth of EAL, Gibson et al.^49^ specifically described that early adelphophagic larvae present a delay in the differentiation of the endoderm- and mesoderm-derived tissues^50^. Therefore, at this point in their development, the heterochrony observed in adelphophagic larvae may be related to the high expression of the thyroid hormone receptor β (THB) gene (Table 2). In echinoderm species, it has been demonstrated that the thyroid hormone (TH) accelerates development to metamorphosis^51^. Typically, larvae obtain TH through consumption of microalgae; however, the complete intracapsular development of EAL indicates that these larvae may produce TH by themselves or could obtain it from nurse eggs or even via cannibalism^10^.

Lastly, another group of DEGs that could be particularly important in EAL of *B. wellingtonensis* are HLH6, t-bx2, and t-bx20 (Supplementary File S4). HLH6 is a transcription factor in *C. elegans* that acts as a development regulator in the cells of the pharyngeal gland and is necessary for feeding^52^. The deletion of HLH6 in this species produces a phenotype similar to that observed in starvation conditions (slow growth rate and small size)^52^. The t-bx2 gene regulates the development of the pharyngeal muscles^53^, and t-bx20 plays a role in the development of the hindgut^54^. Chowdhuri et al. observed arrested development in tbx-2 mutant larvae of *C. elegans* associated with an affected pharynx that made ingestion impossible^53^. In annelids, t-bx2 is expressed in the dorsal side of the embryo^55^, and in more advanced stages of development, the expression of t-bx2 is clearly associated with the intestine and the pharynx^56^. This suggests a conserved expression of the HLH6 and tbx genes during the development of the intestine and the pharynx in invertebrates. Additional research on these genes will be required to understand their evolution and function in polychaetes’ larval feeding patterns.

In conclusion, our results indicate that the different larval trophic modes of *B. wellingtonensis* present transcriptomic plasticity related to changes in the expression of specific genes that could be related to the evolution of larval developmental modes in marine invertebrates. These genes could be tested in future studies by modifying their nutrition sources and lab-controlled environmental factors, and results could be refined as more functional data are accumulated. Comparative RNAseq analyses to other species displaying similar reproductive modes could be a productive avenue in distinguishing whether these differences are a product of adaptive evolution specific of *B. wellingtonensis* or are due to the evolutionary history of poecilogonous species.

## Methods

### Sampling locations and sample processing

Samples for transcriptome sequencing were collected from two localities in Chile: Coliumo (36° 33 11 S; 72° 57 24 W) and Los Molinos (39° 50 46 S; 73° 23 54 W). Samples were found in the high intertidal zone perforating sedimentary rock. They were transported alive to the laboratory and processed within 24 h. Individuals and larvae were classified under a binocular stereo microscope. For subsequent analyses, the samples were classified into seven groups: Type I planktotrophic larvae (PLTI), Type III planktotrophic larvae (PLTIII), early adelphophagic larvae (EAL), late (juvenile) adelphophagic larvae (AL), Type I Female (FTI), Type III Female (FTIII), and male (M). Each group contains three biological replicates. Determination of the developmental stage of the larvae was based on the number of setigers (segment bearing chaetae), a method commonly used in polychaetes^13^. Larvae of the groups EAL, PLTI, and PLTIII used for the experiment had 5 setigers while the AL had at least 10 setigers. Adelphophagic larvae with less than 5 setigers cannot be differentiated from PLTIII. Supplementary File S5 depicts a summary of the larval development and reproductive strategies of *B. wellingtonensis*. In order to have sufficient tissue for the analyses, larvae were collected from capsules of different mothers and pooled depending on their trophic type and developmental stage.

### RNA extraction

Samples were homogenized in QIAzol Lysis Reagent (Qiagen). Total RNA extraction was performed using the Universal RNeasy Mini Kit with a gDNA Eliminator Solution (Qiagen) according to the manufacturer’s instructions. We used a NanoDrop 2000 Spectrophotometer (NanoDrop Technologies) and Fragment Analyzer to assess quantity, purity, and integrity of the RNA samples. All samples used for subsequent analyses had to have RIN > 8, as previously described^14,57^.

### Illumina sequencing and data quality control

Starting with 100 ng of RNA from each sample library, building (with individual barcodes) was performed with the KAPA Stranded mRNA-Seq Kit Illumina Platforms (KappaBiosystem) according to manufacturer’s instructions. The quality of the library was checked in Qubit 2.0, Agilent 2100, and Q-PCR. After passing the quality filters, the libraries were pooled according to their effective concentration and sequencing into HiSeq 4000 by Macrogen of Seoul, Korea (http://www.macrogen.com); this process yielded read lengths of 100 nt. Data quality was checked at Macrogen and provided in FASTq format. We obtained a total of 1,157,058,616 PE reads. The raw data files were deposited into the Short Read Archive (SRA) of NCBI (BioProject PRJNA666738).

### Assembly and annotation

The sequenced raw data was processed using the CLC Genomics Workbench software v.12.0.3 (Qiagen Bioinformatic) in a four-step pipeline: (i) removal of adapter sequences, (ii) removal of ambiguous nucleotides (‘N’), (iii) trimming base pairs with a Phred quality score 0.001 from the 3′-end of each sequence, and (iv) removal of reads shorter than 50 bp after trimming. The trimmed reads were subjected to a quality assessment using FastQC and MultiQC. High quality reads were assembled using the CLC de novo assembly option, and the following parameters were applied: mapping mode = Map read back to contig (slow), bubble size = 93, minimum contig length = 200 nt, word size = 25, perform scaffolding = yes, auto-detect paired distances = yes. The quality and completeness of the new draft assemblies were analyzed using the software QUAST for assembly statistics^58^. Moreover, comparisons were made to the Benchmarking Universal Single-Copy Orthologs (BUSCO) database using the Metazoan (metazoa_odb9), which consists of 978 single-copy genes that are present in at least 90% of Metazoan^59^.

TransDecoder v5.3.0 was used to identify putative protein coding regions, and homology options were included as retention criteria for the candidate ORFs. Predicted ORFs were used for BLAST searches and annotation against the UniProt (Swiss-Prot) and Uniref90 using the BLASTX algorithm with an e-value cutoff of 1e−10. Annotated unigenes were further searched for Gene Ontology (GO) terms using the Blast2GO software in the main GO categories of molecular functions, biological processes, and cellular components^60^. Complementary annotations were done with the InterProScan v.5 software, which provides functional analysis of proteins by classifying them into families and predicting domains and important sites. The annotation results were further fine-tuned with the EggNOG and GO slim functions of the Blast2GO software in order to improve and summarize the functional information of the transcriptome dataset. Supplementary File S6 depicts the bioinformatics workflow used in this study.

### Differential gene expression analysis

Trimmed reads of each sample were mapped back separately on the high-quality set transcripts by applying the following parameters (mismatch cost = 2, length fraction = 0.8, similarity fraction = 0.8 and maximum number of hits for a read = 10) and using the RNA-Seq tool of the CLC Genomics Workbench v12.0.3 (Qiagen Bioinformatic). Thus, the expression of each transcript in each sample was calculated as Transcript Per Million (TPM). Subsequently, a DEA between adults, larval samples, and intro larvae was carried out in order to identify DEGs using the Differential Expression for RNA-Seq tool of the CLC Genomics Workbench v12.0.3 (Qiagen). The normalization factors were calculated using the trimmed mean of M-values (TMM) method^61^. The threshold FDR < 0.01 was adjusted to identify the differentially expressed genes by log2 fold change [≥ 1].

### Gene ontology and enrichment analysis

A GO enrichment analysis was conducted to reveal GO terms significantly enriched (*p* value < 0.01) in DEGs using the CLC Genomics Workbench v12.0.3 (Qiagen). The redundant enriched GO terms were removed using the web server REVIGO (http://revigo.irb.hr)^62^ The SimRel semantic similarity was used with small-allowed similarity (0.5), and all other parameters were kept as default. Resulting .cvs tables were exported and used to graphically plot the condensed GO terms using ggplot2 in Rstudio^63^.

## Supplementary Information

Supplementary Information 1.

Supplementary Information 2.

Supplementary Information 3.

Supplementary Information 4.

Supplementary Information 5.

Supplementary Information 6.

Supplementary Legend.
