# Supplementary figures and images for: RNA-Seq reveals divergent gene expression between larvae with contrasting trophic modes in the poecilogonous polychaete Boccardia wellingtonensis
Source: Sci Rep. 2021 Jul 22;11:14997. doi: 10.1038/s41598-021-94646-y (PMC8298564; doi:10.1038/s41598-021-94646-y)

Length distribution

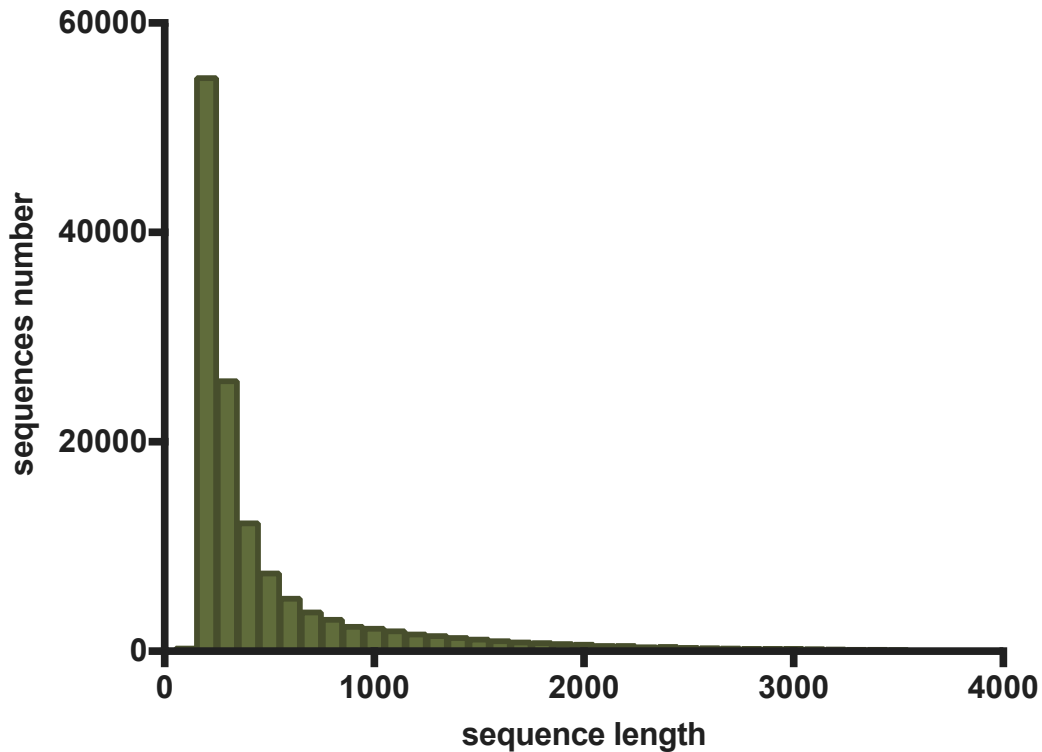

Supplement: Supplementary file 2 — Supplementary Information 2. [file 41598_2021_94646_MOESM2_ESM.pdf]

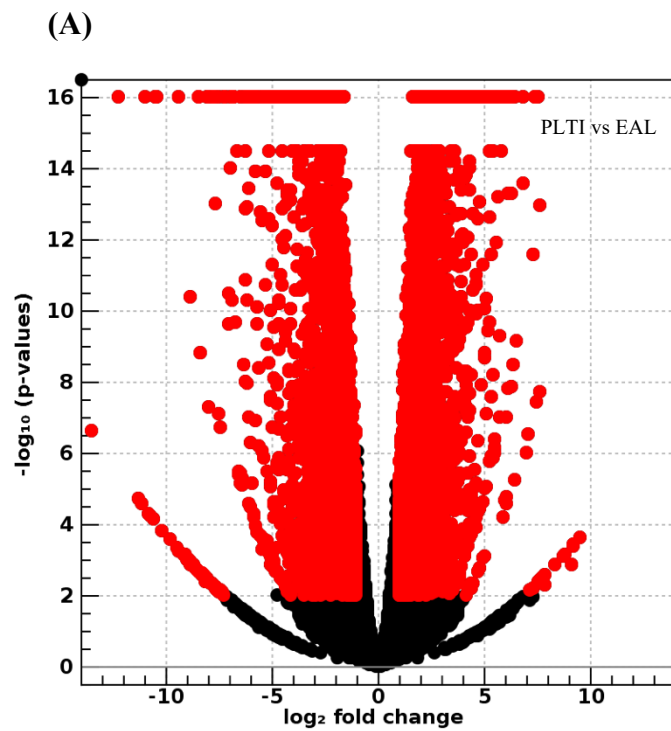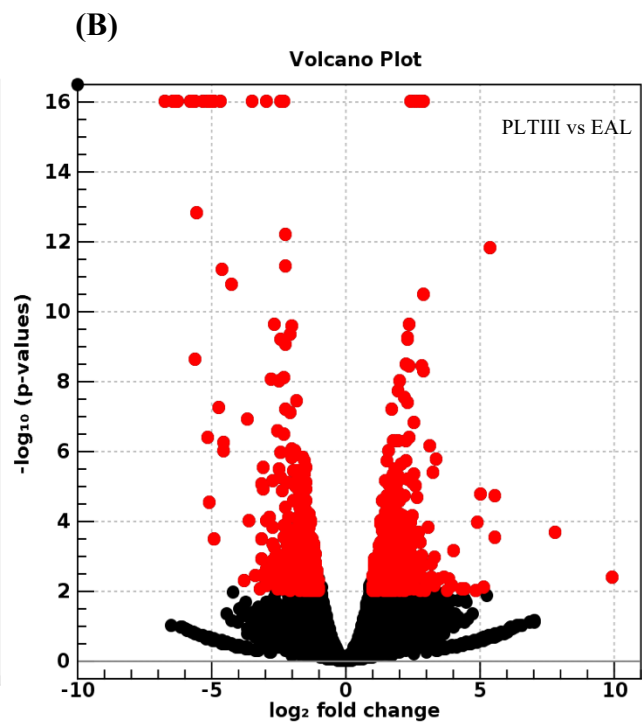

(C)

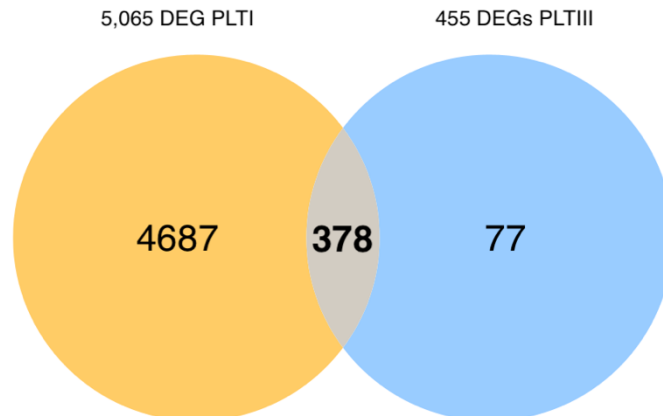

Supplement: Supplementary file 3 — Supplementary Information 3. [file 41598_2021_94646_MOESM3_ESM.pdf]

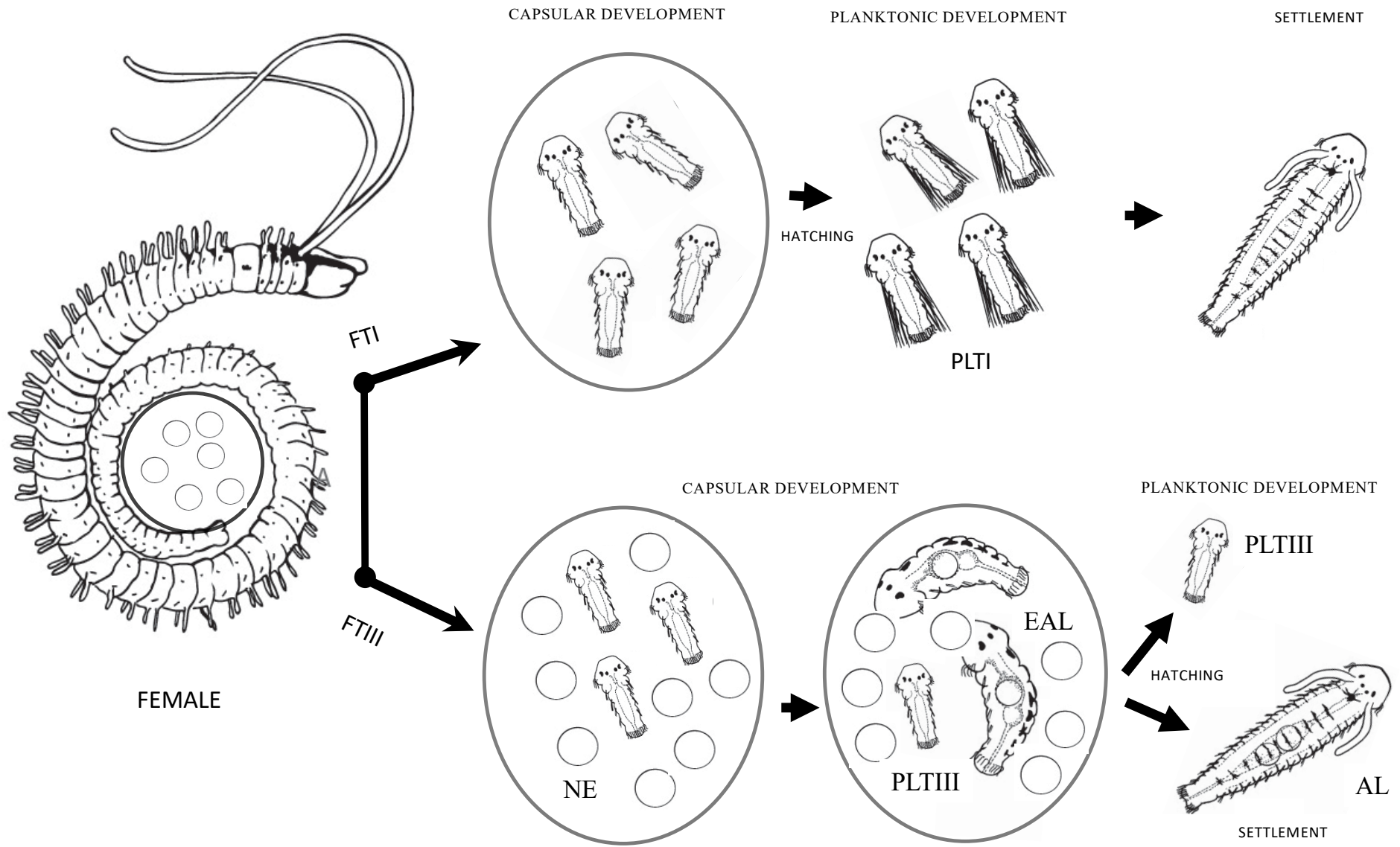

Supplement: Supplementary file 5 — Supplementary Information 5. [file 41598_2021_94646_MOESM5_ESM.pdf]

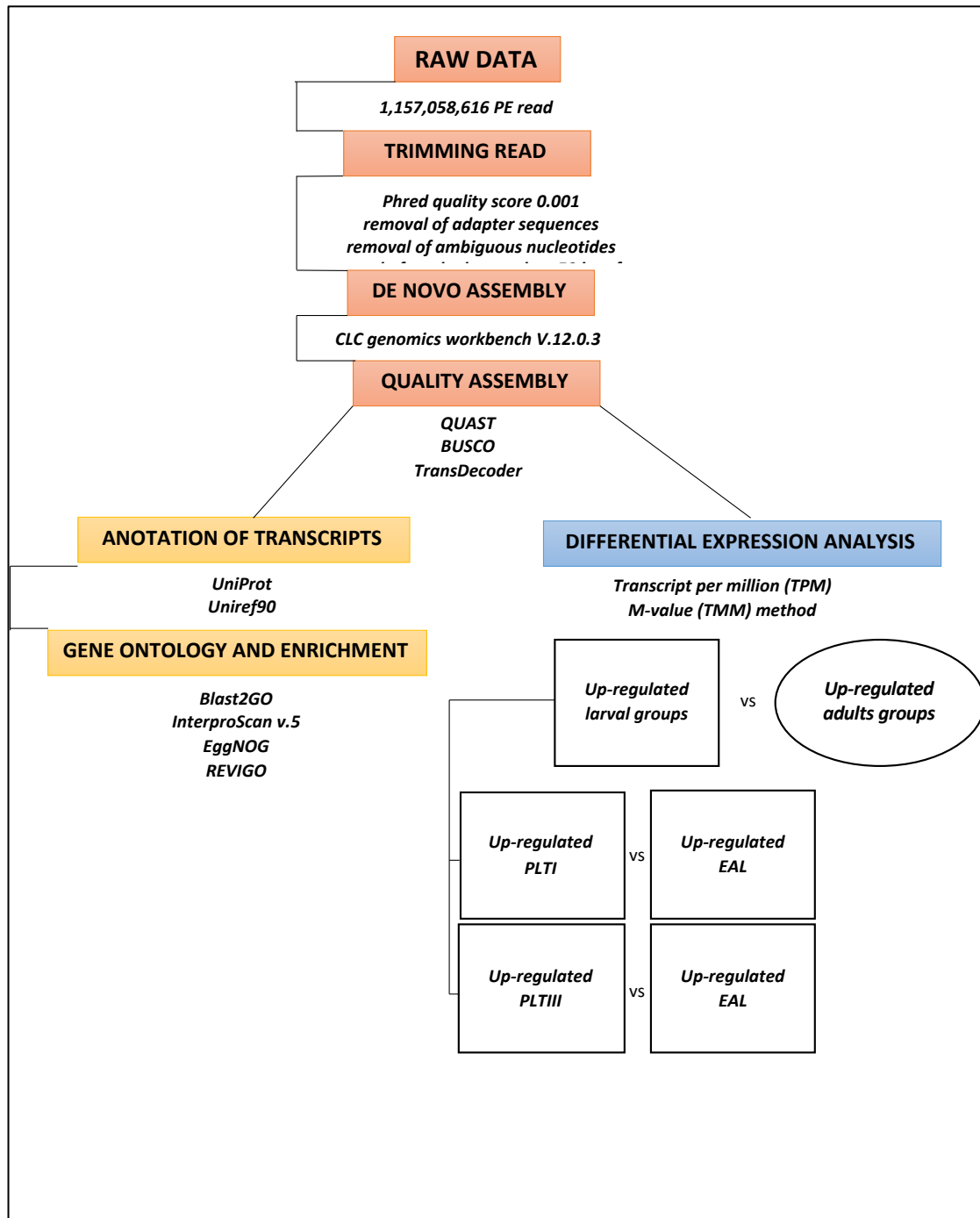

Supplement: Supplementary file 6 — Supplementary Information 6. [file 41598_2021_94646_MOESM6_ESM.pdf]
