# Supplementary material for: RNA-Seq reveals divergent gene expression between larvae with contrasting trophic modes in the poecilogonous polychaete Boccardia wellingtonensis
Source: Sci Rep. 2021 Jul 22;11:14997. doi: 10.1038/s41598-021-94646-y (PMC8298564; doi:10.1038/s41598-021-94646-y)
Supplement: Supplementary file 7 — Supplementary Legend. [file 41598_2021_94646_MOESM7_ESM.docx]

**Supporting Information**

**RNA-Seq reveals divergent gene expression between larvae with contrasting trophic modes in the poecilogonous polychaete *Boccardia wellingtonensis***

Álvaro Figueroa^1^, Antonio Brante^2,3^, Leyla Cárdenas^1,4^

^1^Instituto de Ciencias Ambientales y Evolutivas. Universidad Austral de Chile

^2^Centro de Investigación en Biodiversidad y Ambientes Sustentables, Facultad de Ciencias. Universidad Católica de la Ssma. Concepción

^3^Departamento Ecología. Facultad de Ciencias. Universidad Católica de la Santísima Concepción, Concepción, Chile.

^4^Centro Fondap-IDEAL

Corresponding author(s): Álvaro Figueroa (alvaroalejandrofigueroa@gmail.com)

**CONTENT**

**-Supplemental File S1.** Number of retained reads and average length after that reads were subjected to a quality trim (Phred = 0.001) and adapters removal.

-**Supplemental File S2**. The length and distribution of the assembled transcripts of *B. wellingtonensis* transcriptome.

-**Supplemental File S3**. (A–B) Volcano plots showing differentially regulated genes over-represented in the larval group with different trophic modes. Red dots highlight differentially expressed genes with threshold FDR < 0.01 and Log2 Fold Change [≥1]. The black zone indicates the number of transcripts that do not show significant differential expression. (A) PLTI vs EAL; (B) PLTIII vs EAL. (C) Venn diagram representing commonly up‐regulated genes (378) in both planktotrophic groups (PLTI and PLTIII) in comparation with EAL.

-**Supplemental File S4**. Genes whose expression was significantly different between larvae and adults, between adult females (FTI vs. FTIII) and between larval samples with different trophic modes (PLTI vs EAL; PLTIII vs EAL) with their functional annotations. Details of the GO terms enriched in Biological Process (BP), molecular functions (MF) and cellular components (CC) in planktotrophic and adelphophagic larvae.

**- Supplemental File S5.** Schematic representation of development the different larval trophic modes in *Boccardia wellingtonensis*

-**Supplemental File S6**. Schematic diagram of bioinformatics workflow used in this study
